# Determining multiallelic complex copy number and sequence variation from high coverage exome sequencing data

**DOI:** 10.1186/s12864-015-2123-y

**Published:** 2015-11-02

**Authors:** Diego Forni, Diana Martin, Razan Abujaber, Andrew J. Sharp, Manuela Sironi, Edward J. Hollox

**Affiliations:** Department of Genetics, University of Leicester, Leicester, UK; Bioinformatics, Scientific Institute IRCCS E.MEDEA, Bosisio, Parini, Italy; Department of Genetics and Genome Sciences, Icahn School of Medicine at Mount Sinai, New York, NY USA

**Keywords:** Exome, High throughput sequencing, Copy number variation, Beta-defensin

## Abstract

**Background:**

Copy number variation (CNV) is a major component of genomic variation, yet methods to accurately type genomic CNV lag behind methods that type single nucleotide variation. High-throughput sequencing can contribute to these methods by using sequence read depth, which takes the number of reads that map to a given part of the reference genome as a proxy for copy number of that region, and compares across samples. Furthermore, high-throughput sequencing also provides information on the sequence differences between copies within and between individuals.

**Methods:**

In this study we use high-coverage phase 3 exome sequences of the 1000 Genomes project to infer diploid copy number of the beta-defensin genomic region, a well-studied CNV that carries several beta-defensin genes involved in the antimicrobial response, signalling, and fertility. We also use these data to call sequence variants, a particular challenge given the multicopy nature of the region.

**Results:**

We confidently call copy number and sequence variation of the beta-defensin genes on 1285 samples from 26 global populations, validate copy number using Nanostring nCounter and triplex paralogue ratio test data. We use the copy number calls to verify the genomic extent of the CNV and validate sequence calls using analysis of cloned PCR products. We identify novel variation, mostly individually rare, predicted to alter amino-acid sequence in the beta-defensin genes. Such novel variants may alter antimicrobial properties or have off-target receptor interactions, and may contribute to individuality in immunological response and fertility.

**Conclusions:**

Given that 81 % of identified sequence variants were not previously in dbSNP, we show that sequence variation in multiallelic CNVs represent an unappreciated source of genomic diversity.

**Electronic supplementary material:**

The online version of this article (doi:10.1186/s12864-015-2123-y) contains supplementary material, which is available to authorized users.

## Background

Copy number variation (CNV), where a section of DNA differs from a diploid copy number of 2 between different individuals, is a common form of variation that can affect a substantial portion of the human genome and genomes from other organisms [1–3]. Such variation can affect phenotype through a variety of mechanisms, such as a gene dosage effect [4], variation in the number of protein coding domains [5] or alteration of position of enhancer elements [6]. CNV can be divided into simple CNV, usually comprising a deletion or duplication and generated by one mutational event, and complex multiallelic CNV where different dosage variants are generated by recurrent mutation. In humans, complex CNV has been shown to affect disease susceptibility. For example, CNV of the beta-defensin locus has been shown to affect the risk of developing the inflammatory skin disease psoriasis [7, 8], and CNV of the neuronal glucose transporter *SLC2A3* modifies the age of onset of Huntington’s disease [9]. Furthermore, CNV of the amylase gene in dogs has been shown to be an adaptation to domestication and in Drosophila CNV is important in resistance to insecticides [10, 11].

Despite the importance of complex CNV, research progress has lagged behind studies into simpler deletions and duplications, and has been dogged by false positive disease associations. This is almost entirely due to the challenges in accurately typing complex multiallelic CNV [12]. Most studies have used real-time quantitative PCR, which is prone to bias [13, 14]. Although other more reliable methods exist, such as the paralogue ratio test (PRT) and multiplex amplifiable probe hybridisation (MAPH), such assays require extensive validation and are locus specific rather than genome-wide, limiting the amount of data generated at one time. Genome-wide approaches, such as analysis of hybridisation signal intensities from SNP chips and array comparative genomic hybridisation, generate a large amount of useful data but are often weak in typing complex CNV – i.e. giving an absolute copy number rather than just indicating a loss or gain of signal. Furthermore, complex CNV detection and typing appears to be DNA cohort dependent, which further increases the risk of false negative genetic associations and false positive associations due to batch effects.

Recent work has addressed analysis of CNVs using sequence read depth (SRD) generated by next-generation sequencing platforms [4, 15]. The principle is simple: when short read sequences are generated from an individual with a high copy number for a particular region and are subsequently aligned to a reference genome with only one copy of that region, the number of reads mapping to that region will be higher than expected. Most studies have focused on detecting CNV in whole genome sequences rather than typing absolute copy number of particular complex CNV regions [16]. One exception relies on singly-unique-nucleotides (SUNs) to provide an internal calibration of copy number of that particular region [15]. This has been successful for some CNVs, and using this approach the role of a particular sequence variant of the CNV carrying the *SRGAP2* gene in human evolution has been elucidated [17]. Nevertheless, the extent to which other complex CNVs show that particular pattern of variation, where SUNs exist and are frequent, is not clear.

One distinct advantage of SRD approaches is that they can yield information on the sequence differences between different copies of the complex CNV. Other methods, such as analysis of SNP array hybridisation intensity data, array comparative genomic hybridisation (aCGH) data, MAPH or PRT often assume sequence identity under the probe or primer. The effect of this can be minimised either by multiple assays or careful design of probes/assays, but such methods remain essentially blind to the sequence variation between copies. This sequence variation can be important, for example, in the *SRGAP2* example mentioned above, where only particular paralogues, defined by sequence variants, are functional [17].

An accurate assessment of sequence variation of a multiallelic CNV locus using SRD needs high coverage sequence data to be confident that all copies have their sequence represented in the alignment. The number of publically-available whole genomes sequenced at high coverage is increasing but limited, but the 1000 Genome Project has released sequence data of 2455 exomes sequenced at high coverage using Illumina sequencing technology. Furthermore, given the lower cost of exome sequencing per sample, exome sequences are becoming increasingly common for large sample sets. Therefore we focused on complex CNV typing and sequence analysis using high coverage exome sequence data.

There are several platforms that use exome SRD data to detect and call CNV [16]. All use a similar approach of using principal component analysis to extract components describing different aspects of the variation, including noise, and then using a hidden Markov model approach to call boundaries between gain of copy number, loss of copy number and diploid regions. In principle, the variation in the principal component that most closely corresponds to DNA dosage signal can be used to type the absolute integer copy number of complex CNV. Only one platform has been used to do this, CoNIFER, which has been used on 907 exomes to show proof of principle [18], and subsequently on 1644 exomes to investigate the role of CNV in autism spectrum disorder [19].

To rigorously test the quality of copy number and sequence variation calls from exome data on a region of complex CNV, we decided to focus on the well-studied human beta-defensin locus. This is a 322 kb region of DNA on chromosome 8p23.1, which is embedded within a complex region rich in segmental duplications and olfactory receptor genes [20, 21]. The 322 kb region is copy number variable as a block, with the diploid copy number commonly between 2 and 7 copies, but individuals with as few as one copy and as many as 12 copies have been observed [14, 22]. Chromosomes with high copy number of the beta-defensin region can be distinguished by a visibly larger 8p23.1 region by G-band staining [23]. Furthermore, the beta-defensin region is polymorphic in physical location on the chromosome, with some copies polymorphically at the proximal end of 8p23.1 within the olfactory repeat region REPP in addition to the distal end within the olfactory repeat region REPD [20].

Seventeen annotated genes are within the copy number variable region, including eight beta-defensin genes (*DEFB4*, *DEFB103*, *DEFB104*, *DEFB105*, *DEFB106*, *DEFB107, DEFB108* and *DEFB109*) and nine other genes mostly expressed in the testes [21]. The role of *DEFB4* and *DEFB103* appear to be immunomodulatory in humans, in addition to direct antimicrobial activity. For the other defensins, the role is less clear, although a knockout mouse deleting the orthologous beta-defensin cluster shows complete male infertility, strongly suggesting an important role of these genes in reproduction [22]. The function of the other genes remains unknown. Sequence variation of the region remains understudied, primarily due to the difficulty of reliably distinguishing variant nucleotides in multicopy regions by the Sanger sequencing method. We therefore decided to test whether exome SRD could accurately call copy number and sequence variation of this complex CNV, both to identify beta-defensin variants which may have novel functions but also to robustly establish a recommended approach that could be applied to complex CNVs by the research community.

## Methods

### Exome data

Filtered phase 3 exome data generated by the 1000 Genome project was downloaded from the European Bioinformatics Institute (http://ftp.1000genomes.ebi.ac.uk/vol1/) as fastq files. We retrieved only Illumina technology filtered paired end data generated by four different sequencing centres: Baylor College of Medicine (BCM), Beijing Genomic Institute (BGI), Broad Institute (BI), and Washington University Genome Sequencing Center (WUGSC). The initial dataset consisted of 2455 samples divided in 5 major continental groups (referred to as “super populations” by the 1000 Genomes Project): African, East Asian, South Asian, European, and Admixed American. Throughout this paper, the standard abbreviations for 1000 Genomes populations are used – see http://www.1000genomes.org/category/frequently-asked-questions/population for details of population codes and sample names.

### Mapping

The reference sequence was generated using all chromosome 8 exons plus 300 bp flanking sequence from the GRCh37-hg19 human reference genome; when two exons were closer than 600 bp, all the genomic distance was considered. We used mrsFAST ultra v3.2.0 for sequence alignment, using a paired end approach [24]. Importantly, ConIFER was designed to be used on alignments generated using mrsFAST [25], a one-to many sequence read aligner, rather than other more common aligners based on the Burrows-Wheeler algorithm that generally operate as a one-to-one sequence aligner. mrsFAST therefore captures all possible mapping locations up to a user-defined number of mismatches, which leads to more accurate estimates of copy number from SRD approaches, because all copies above a given percentage identity will be aligned efficiently to the reference locus. A reference indexing window size of 12 was created and the pair end reads mapping was performed using the all-mapper tool with a maximum error threshold of 6.

### CNV calling

CNV calling used CoNIFER, a suite of Python scripts that calculate RPKM (reads per thousand bases per million reads sequenced) values starting from aligned sequences and a set of probes [18]; for our analyses we defined the probe set as all the chr8 exon boundaries. Samples with a RPKM mean <50 were discarded from the analysis to reduce background signal. Singular value decomposition standardized z-scores RPKM (SVD-ZRPKM) values were calculated for samples belonging to one or more population generated by the same sequencing centre (e.g. all African samples from BGI), removing the first components that are disproportionally responsible for the variance of the data. For each dataset under analysis, we evaluated the best number of components to be removed using scree plots generated by CoNIFER; the components to be removed were chosen based on the shoulder of the scree plot. Raw SVD-ZRPKM mean of the beta-defensins that unambiguously map just to chromosome 8p23.1 *DEFB4*, *DEFB103*, *DEFB104*, *DEFB105*, *DEFB106* and *DEFB107*) were retrieved and used for CNV calling. To determine the integer copy number call for each sample we used CNVtools, grouping samples from the same continental group and sequencing centre together. The Gaussian mixture models were evaluated by eye, plotting the mixture model together with a histogram of raw copy number to check for clear clustering about integer copy numbers, and also by comparing calls with previous copy number estimates of samples from the HapMap collection that overlapped the 1000 Genomes dataset, using triplex paralogue ratio tests [26]. These positive controls also allowed us to confirm that the correct SVD-ZRPKM component was used for copy number calling.

### Variant calling

Single nucleotide variants in one assembled beta-defensin region were called using FreeBayes v9.9.2 [27]. Freebayes is a haplotype-based Bayesian genetic variant detector, which can call sequence variants from samples of different ploidy, with ploidy (equivalent to, in our case, copy number) as an extra parameter for each sample. We ran FreeBayes setting 30 and 50 as minimum base and mapping quality, respectively; 0.10 as the minimum fraction of alternate variant observations and 10 for the minimum count of alternate variant reads; finally we specified for each sample the number of copies of the beta-defensin region estimated by the CoNIFER exome SRD analysis.

### Validation of copy number

Copy number estimates of 164 samples from the HapMap collection that overlapped the 1000 Genomes dataset, using triplex paralogue ratio tests, have been published previously. Copy number estimates of the same samples were also obtained using the Nanostring nCounter system [28], by designing 6 probes mapping to the beta-defensin repeat and calculating the first principal component, sample-wise, of the 6 probe values using the R package CNVtools [29].

### Validation of sequence variants

PCR primers were designed flanking variable sites, and genomic DNA from a selection of 8 HapMap samples amplified using standard PCR. PCR products were then cloned into pJET 1.2 vector using the CloneJet PCR cloning kit (Thermo Scientific), where the insert disrupts a lethal gene allowing only plasmids with an insert to confer viability to the bacterial host. Following plating, colonies were selected for colony PCR using the vector primers 5’CGACTCACTATAGGGAGAGCGGC-3’ and 5’AAGAACATCGATTTTCCATGGCAG-3’ flanking the insert, according to the manufacturer’s instructions. PCR products were then digested using the appropriate restriction enzyme to distinguish the alleles at the variant site (*Msp*I for rs140952426, *Ape*KI for rs200757797) and scored for allelic state following agarose gel electrophoresis.

### Population genetics

Variant frequency distributions for non-synonymous and synonymous variants were compared across the beta-defensin genes analysed, for each population, using the k-sample Anderson-Darling test [30] implemented by the R package kSamples.

### Availability of data

One thousand Genomes phase 3 exome read data are publically available from the European Bioinformatics Institute European Bioinformatics Institute (http://ftp.1000genomes.ebi.ac.uk/vol1/) as fastq files. All 1285 confident copy number calls have been submitted to dbVar (http://www.ncbi.nlm.nih.gov/dbvar) with accession number nstd116. All novel variants identified in this paper have been deposited in dbSNP (http://www.ncbi.nlm.nih.gov/SNP/).

### Ethics statement

For a description of the ethics and consent agreements used for the 1000 Genomes Project see http://www.1000genomes.org/about.

## Results

**Fig. 1:**
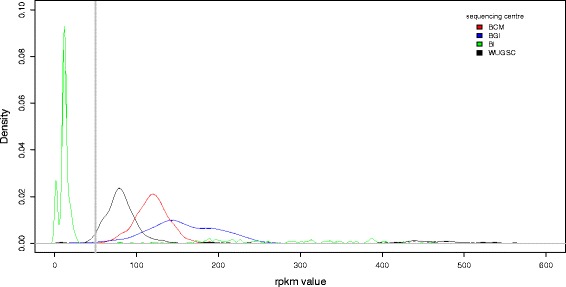
Distribution of reads-per-kilobase-per-million-reads (RPKM) values of different samples stratified by sequencing centre. The kernel density plot shows density of RPKM values from mrsFAST alignments for four different sequencing centres, distinguished by the different colours. The vertical dotted line indicates the cutoff value at RPKM = 50, with samples above that threshold taken on for copy number calling

**Fig. 2:**
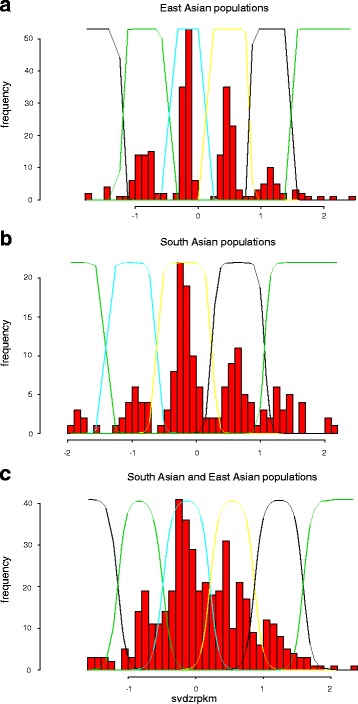
Effects of continental group batch of origin on copy number clustering. The histograms show normalised sequence depth coverage data for the beta-defensin region generated by the BGI sequencing centre. X-axis values represent raw mean SVD-ZRPKM values, and the y-axis represents number of samples. Curved lines indicating the Gaussian curves used to call integer copy number. **a**) samples from East Asian populations (*n* = 269), **b**) samples from South Asian populations (*n* = 165), **c**) samples from South Asian and East Asian populations analysed as one batch (*n* = 434)

**Fig. 3:**
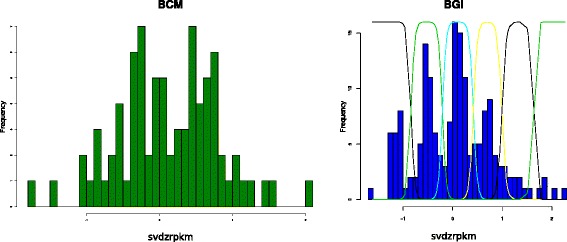
Effects of sequencing centre and batch size on copy number clustering. The histograms show normalised sequence depth coverage data for the beta-defensin region for sub-Saharan African samples. X-axis values represent raw mean SVD-ZRPKM values a) BCM sequencing centre, *n* = 81 (15 YRI, 57 LWK, 9 ASW). b) BGI sequencing centre, *n* = 172 (26 YRI, 3 LWK, 25 GWD, 43 MSL, 47 ESN, 5 ASW, 23 ACB), with curved lines indicating the Gaussian mixture model used to call integer copy number

We aligned fastq raw sequence read files for 2455 samples from 26 populations to a concatenated reference sequence made of all chromosome 8 exons plus 300 bp flanking sequence. We grouped the samples from the 26 populations into four continental groups. After removing samples where the reads per kilobase per million mapped reads (RPKM) mean value was less than 50, (Fig. 1, Table 1) we calculated single variant decomposition scores batch wise, with each batch representing a distinct continental group/sequencing centre combination. Raw SVD-ZRPKM mean of all exons of the beta-defensin genes were retrieved and a Gaussian mixture model fitted for each set of data using CNVtools. Observing clear clustering of SVD-ZRPKM mean values about integer copy number values leads us to have high confidence in the Gaussian mixture model fit and therefore in the final copy number calls. Importantly, batches needed to be defined by both sequencing centre and continental group, otherwise poor clustering was observed. For example, Fig. 2 shows histograms of raw SVD-ZRPKM mean values from the BGI sequencing centre, for East Asians (Fig. 2a), South Asians (Fig. 2b) and East and South Asians together (Fig. 2c). Although a Gaussian mixture model can be fitted for all three histograms, the clustering of the SVD-ZRPKM values of the combined batches (Fig. 2c) is visibly less distinct than when each batch is analysed separately. For most batches, clear clustering of raw SVD-ZRPKM values was observed, increasing confidence that the correct copy number was being called (Fig. 3a). However some showed SVD-ZRPKM values that did not cluster well (e.g. African samples from BCM sequencing centre, Fig. 3b), and these were removed from subsequent analyses.

**Table 1:** Characteristics of exome sequences analysed

Centre name	Samples	Samples with > 50 reads per exon	Sample with a copy number call	Samples with a copy number call *P* > 0.95*	Sequence enrichment method
BCM	228	228	147	129 (88 %)	HSGC VCRome custom array
BGI	894	866	866	795 (91 %)	NimbleGen SeqCap_EZ_Exome v2
BI	830	161	161	140 (87 %)	Agilent SureSelect_All_Exon_V2
WUGSC	503	397	288	221 (76 %)	NimbleGen SeqCap_EZ_Exome v3

*posterior probability of the copy number call is >0.95

Gaussian mixture modelling generated a copy number call for each sample with an associated posterior probability of that call. The proportion of calls with a posterior probability greater than 0.95 varied between sequencing centres (Table 1), but overall was 87 %. The distribution of copy number reflected previous results, with 4 being the modal copy number in all continental groups apart from sub-Saharan Africans, and the range of common variation extending from 2 copies to 8 copies per diploid genome (Table 2).

**Table 2:** Beta-defensin copy number frequency in human continental groups

Continental group	Total Samples	2 copy	3 copy	4 copy	5 copy	6 copy	7 copy	8 copy
Sub-Saharan African	214	1 (0.005)	26 (0.12)	60 (0.28)	68 (0.32)	38 (0.18)	16 (0.07)	5 (0.02)
East Asian	405	13 (0.03)	81 (0.200)	153 (0.38)	112 (0.28)	34 (0.08)	10 (0.02)	2 (0.005)
South Asian	248	9 (0.04)	41 (0.17)	97 (0.39)	66 (0.27)	32 (0.13)	3 (0.01)	0
European	349	7 (0.02)	50 (0.14)	142 (0.41)	109 (0.31)	35 (0.10)	6 (0.02)	0
Admixed American	246	6 (0.02)	49 (0.20)	113 (0.46)	64 (0.26)	12 (0.05)	2 (0.008)	0

**Fig. 4:**
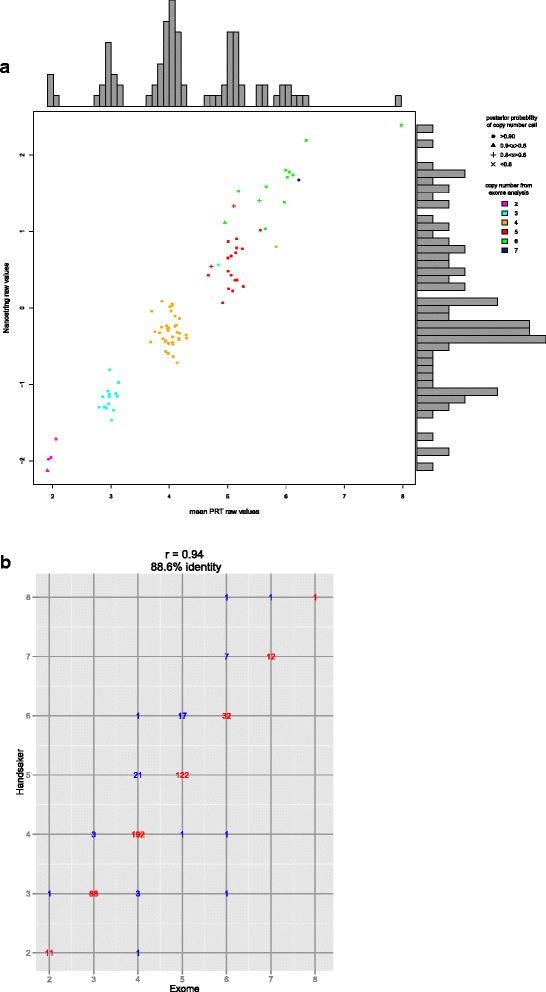
Validated of beta-defensin copy number calling. The plots show comparisons between two methods of calling integer beta-defensin copy number. **a**) comparison with triplex paralogue ratio test and Nanostring nCounter. **b**) comparison with integer calls from phase 1 low coverage whole genome data [4]. The figures in red indicate the numbers of samples concordant for that particular copy number. The numbers in blue indicate the numbers of discordant samples

We validated our copy number calls by comparing calls on a subset of samples with copy number estimates made previously by Triplex PRT [13, 26], by Nanostring [28] (Fig. 4a), and also with copy number calls of the region made by whole genome sequencing [4] (Fig. 4b). It is clear that copy number calls made using exome SRD agree well with both PRT and Nanostring consensus copy number. Exome SRD calls also agree well with whole genome SRD data, although there is a significant discrepancy rate of 11.8 %. Most discrepancies are at the higher copy numbers, and seem to be due to exome SRD underestimating copy number. Of the 7 samples that are discrepant between exome SRD and both PRT and Nanostring, three are also discrepant with whole genome SRD copy number calls, all are spread across the different sequencing centres (Table 3), suggesting the discrepancies are due to random assay noise rather than a systematic bias.

**Table 3:** Discrepant copy number calls between exome sequence read depth (SRD) analysis and previous analyses

Sample	Pop	Batch	Posterior probability of Exome SRD call	Copy number from Exome SRD	PRT and nanostring copy number	Copy number from whole genome SRD
NA18858	YRI	BGI	0.589	6	8	8
NA18861	YRI	BGI	0.996	5	6	6
NA11892	CEU	BGI	0.968	3	5	3
NA11893	CEU	BGI	0.977	4	5	5
NA12156	CEU	BCM	0.610	5	6	na
NA18912	YRI	BI	0.982	6	5	6
NA12761	CEU	WUGSC	0.995	4	3	na

**Fig. 5:**
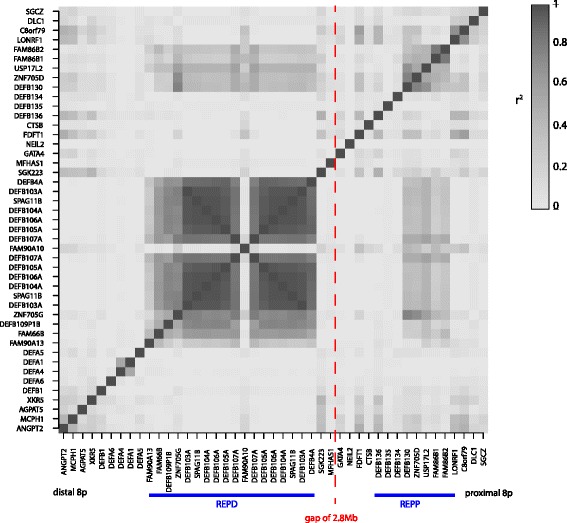
Correlation of SVD-ZRPKM values between genes at 8p23.1. Plot of pairwise correlation between SVD-ZRPKM values among genes at chromosome region 8p23.1. The SVD-ZRPKM mean for all exons belonging to each gene was calculated and the pairwise correlation for each pair of genes was evaluated by the r^2^ metric (the correlation is increasing with gray shading). Gene presence and location is based on the annotation of the hg19 human genome assembly. Complex repeat-rich regions REPP and REPD are indicated, and several genes between REPP and REPD are omitted to save space, as indicated by the red dashed line

We used our exome SRD data to investigate the extent of contiguous copy number variation at this locus, gene by gene. Individual SVD-ZRPKM mean values of each gene were correlated with the individual SVD-ZRPKM mean values of genes within and surrounding the beta-defensin CNV region, both at distal 8p23.1 (a region called REPD [31]), and proximal 8p23.1 (REPP), across the 171 European samples sequenced at the BGI. We would expect genes on the CNV block to show highly correlated SVD-ZRPKM scores across these individuals, reflecting the CNV. Indeed, the core defensin genes (*DEFB4* to *DEFB107*) showed a very high correlation (Fig. 5) indicating that these are on a contiguous block that shows CNV. This block of highly correlated genes extends distally as far as *FAM90A13* and includes *DEFB109*, albeit with lower correlation coefficients, which is likely to be due to mapping of sequence reads derived from known segmental duplications involving these genes on chromosome 4 and chromosome 12. This confirms the observation made previously using arrayCGH that these genes are involved in the beta-defensin CNV [21], and shows that analysis of exome SRD can be a powerful approach to identify CNV boundaries. Interestingly, a moderate correlation coefficient is observed for some genes at REPP, including *DEFB130* but not *DEFB134*, *DEFB135* nor *DEFB136*. The beta-defensin repeat region (involving *DEFB4* to *FAM90A13*) is not assembled here, but it is known from genetic data that the repeat region can be polymorphically present here at this location [20], and this signal we observe is likely to be due to CNV of the beta-defensin repeat region at REPP.

We used our exome alignment files to call sequence variation across the beta-defensin genes within the CNV. Using FreeBayes, a sequence caller that uses diploid copy number as an extra parameter and therefore can make sequence variant calls from non-diploid regions, we called 436 single nucleotide variants spanning 8811 bp of sequence representing the combined length of the beta-defensin genes. 299 are intronic or intergenic, with 137 within the untranslated regions or coding regions. The majority of variants called are rare or very rare, and are specific to particular continental groups, suggesting that they have arisen very recently in human evolutionary history. 355 variants (81 % of total) are novel and have been submitted to dbSNP.

**Fig. 6:**
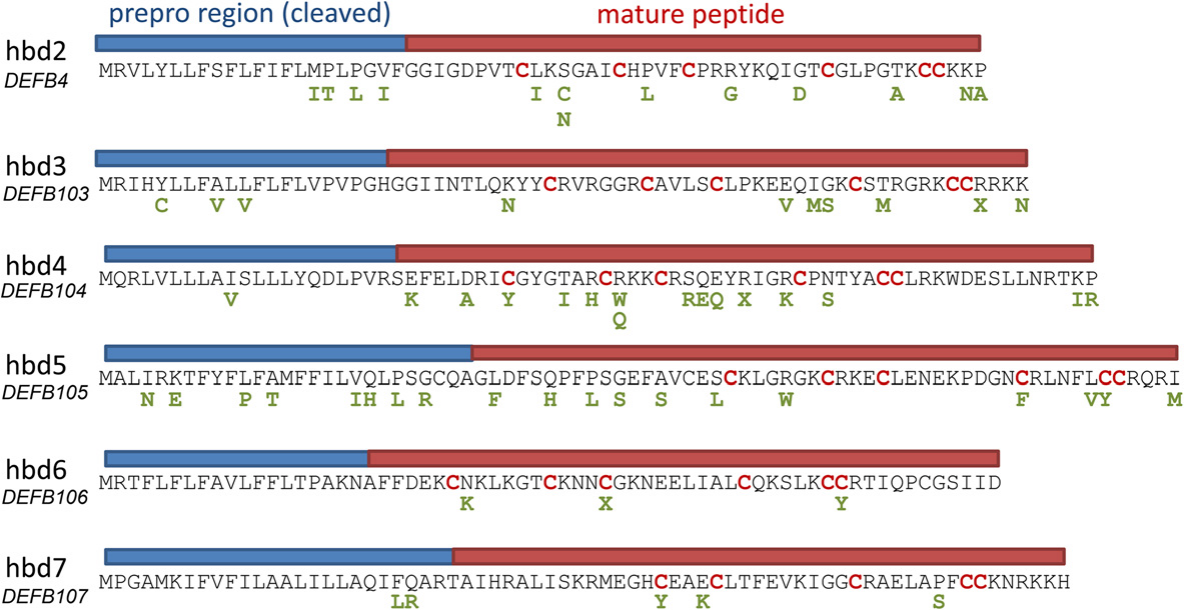
Summary of predicted amino acid changes inferred from sequence variation. The six beta-defensin proteins encoded by the genes analysed in this study are shown. The prepro region, which is cleaved during processing, is shown under the blue bar; with the mature peptide sequence is shown under the red bar. The canonical six cysteines are highlighted in red, with sequence variants identified in this study shown in green. X represents a stop codon, and hbd2, hbd3, hbd4, hbd5, hbd6, and hbd7 are the proteins encoded by *DEFB4*, *DEFB103*, *DEFB104*, *DEFB105*, *DEFB106* and *DEFB107* respectively

Sixty-seven variants (64 non-synonymous substitutions and 3 stop codon gains) were called that were predicted to affect amino acids within the beta-defensin genes (Fig. 6). We validated two frequent non-synonymous variants, rs140952426 in *DEFB104* that changes arginine to a glutamine at position 38, and rs200757797 in *DEFB105* that changes a cysteine to a tyrosine at position 73. It was important not only to validate the presence of the variant but also the correct number of copies of that variant. We did this by amplifying across the variant using genomic DNA, cloning the resulting PCR product, and then counting the number of clones (each derived from a single amplified DNA molecule from the PCR) that had each allele of the variant using colony PCR followed by restriction enzyme digestion (Table 4). This gave an estimate of the proportion of each allele at each variant for each sample, which could be then compared with that predicted by exome sequencing – for example a GGGA genotype (where three copies have a G and one copy has an A at the same paralogous nucleotide site) would be regarded as 0.25 A allele. For both variants, samples homozygous for the variant that is cut by the restriction enzyme was included to provide a background rate of cut failure either due to experimental error or mutation of restriction site during amplification and cloning. The proportion of each allele measured using this approach is consistent with the genotype called from exome SRD data for all samples, except one. The exception is NA12763, where the molecular cloning method generates an estimate which agrees with a copy number of 6 called by PRT rather than a copy number of 4 called by exome SRD, and therefore reflects an error in copy number calling by exome SRD.

**Table 4:** Validation of single nucleotide variants

Sample	rs140952426				rs200757797			
	Uncut (A)	Cut (G)	Proportion A (95 % CI)	From exome	Uncut (T)	Cut (C)	Proportion T (95 % CI)	From exome
NA07056	60	41	0.59 (0.49-0.69)	AAGG	3	84	0.03 (0.01-0.10)	CCCC
NA12044	4	74	0.05 (0.01-0.13)	GGGG	18	53	0.25 (0.16-0.37)	CCCT
NA18956	44	123	0.26 (0.20-0.34)	AGGG	-	-	-	CCCC
NA12004	37	139	0.21 (0.15-0.28)	AGGG	-	-	-	CCCC
NA12156	13	146	0.08 (0.04-0.14)	AGGGG	-	-	-	CCCCC
NA07357	-	-	-	GGGGGG	5	79	0.06 (0.02-0.13)	CCCCCT
NA12763	-	-	-	GGGG	22	178	0.11 (0.07-0.16)	CCCT^a^
NA12874	-	-	-	GGGG	16	52	0.23 (0.14-0.35)	CCCT

95 % confidence intervals of the binomial distribution calculated using the Pearson-Klopper method

^a^Called as 6 copies by previous PRT

We considered whether a signature of selection at these beta-defensin genes could be inferred from the frequency of sequence variants. By comparing the sequence variant frequency distribution of non-synonymous and synonymous SNPs within coding regions, it is possible to detect the effect of negative selection or balancing selection across the region. Assuming that selection does not act on synonymous variants and therefore their variant frequency distribution represents the neutral null model, we would expect to see an enrichment of non-synonymous variants at low frequency under negative selection, and an enrichment of non-synonymous variants at high frequency (0.4-0.5) under balancing selection.

Given the small exon size and therefore small number of polymorphisms in the coding region of each gene, we compared the sequence variant frequency distribution for each continental group separately, combining data across all beta-defensin genes measured in the CNV. We did not find a statistically significant difference between any non-synonymous and synonymous sequence variant frequency distributions, for any of the continental groups. This suggests that selection is not acting on these genes, and that the sequence variants observed are essentially neutral.

## Discussion

In this study we show that high coverage exome sequencing can effectively report integer copy number and sequence variation between copies. Focusing on exome sequencing data has two disadvantages compared to whole genome sequencing data. Firstly, typing depends on the presence of exons (or, more correctly, baits in the exome enrichment mix) that map within the complex CNV region. It is obvious that if there are no exons within the CNV, the sequence will not be enriched and no (or very little) sequence will be generated from that CNV. The problem is scalar, in that it is likely that CNVs with more exonic sequence will have more sequence reads and therefore be more effectively called using SRD approaches compared to CNVs with a small amount of exonic sequence. Secondly, exome sequencing relies on solution-phase enrichment of fragmented DNA using sequence-specific baits. Such an enrichment process might introduce bias into copy number calling, dependent on GC-content of the region or experimental batch, for example.

Future studies need to be aware of several issues that are important to consider when inferring integer copy number from exome SRD. Firstly, in this study we knew a priori the extent of CNV, which had been elucidated previously, so allowing us to choose several genes which we know were contained in a contiguous CNV block. Inferring CNV boundaries from exome data, particularly when those boundaries are within gene sparse areas or segmental duplications, may result in noisier SRD data, although we show that once a CNV can be robustly typed, the boundary of the CNV can be refined on a gene-by-gene basis. Secondly, we had a number of positive controls where copy number was well established using other methods. This allowed us to validate our singular value decomposition components and our Gaussian mixture models, giving us confidence in our final copy number calls. Thirdly, particular population-sequencing centre batches of samples gave distinct raw copy number results, suggesting that fitting individual Gaussian mixture models to individual batches is important. We do not know what causes this variation, and why sometimes it prevents clustering of raw copy number results, but we suspect it might be due to subtly different signal-noise structures in the data because of different exome enrichment processes used by different centres, which SVD cannot completely resolve. If correct, then we predict that high-coverage whole genome sequences will not show this problem.

One previous study used target enrichment and 454 sequencing of over 87 kb of the beta-defensin CNV in two samples to study sequence variation [32]. This study had the advantage of revealing variation in non-coding regions, and, together with the longer reads generated by 454 technology, sequence haplotypes could be inferred. However, CNV calling by read depth was not attempted and some sequence variants, particularly rare variants, were called with a small number of supporting reads, suggesting limited sensitivity of the approach. Furthermore, only two European samples were sequenced, revealing a limited part of the total potential sequence variation of the region.

By comparing sequenced BACs, we have previously shown that sequence differences between different copies is localised particularly immediately upstream and downstream of genes, and this may result in expression differences between different copies of the same gene. Indeed, both luciferase reporter studies in keratinocytes and expression in lymphoblastoid cell lines have shown that variation between different defensin copies affects expression of *DEFB103* and of *DEFB4* [26, 33]. The single nucleotide variation affecting *DEFB103* expression is within 1 kb immediately 5’ of the transcription start site, but the single nucleotide variation affecting *DEFB4* expression is not known, except that it is tagged by a synonymous polymorphism rs2740090, which is reported as common across all the continental groups studied here (Additional file 1: Table S1). Our previous observations of increased sequence differentiation upstream of *DEFB103*, together with its functional effect and the population distribution differences, have led us to suggest that natural selection has influenced expression levels of at least *DEFB103* [26]. Furthermore, given the gene dosage effect of copy number variation observed at this locus [34, 35], gene copy number results in variation in levels of protein which in turn provides a phenotype upon which selection can act. In contrast, in rhesus macaques [21] and dogs [36] it seems likely that amino acid sequence variation between copies has been subject to natural selection.

In Africa, all variants that alter amino acids in the defensin genes are rare, but outside some of these variants have risen to increased frequency. In particular, a variant at *DEFB105 (rs700757797)*, which disrupts the fifth canonical cysteine essential for disulphide bridge formation in the mature protein (Fig. 6), is common in non-African populations (Additional file 1: Table S1). The functional consequences of this and other variants are not known, and await further study. It is likely that they are variants with little effect or null variants, resulting in proteins with reduced function. In the context of a multicopy gene, one or two copies with little or no function will have little consequence, particularly at higher copy numbers, therefore negative selection against such variants is likely to be weak, and indeed we could not detect any signature of selection using variant frequency distributions. An alternative view is that some variants may have alternative off-target effects, and that we have little power to detect selection at a small number of such variants. Indeed given the known receptor promiscuity of defensins [37] and the influence of structural changes on antimicrobial activity [38, 39] this is a possibility worth exploring in the context of novel therapeutic agents.

## Conclusions

We have used exome sequence data from the 1000 Genomes Project to call copy number for the human beta-defensin region on 1285 individuals. We have also identified 436 sequence variants that differ between copies and between individuals, mostly rare, of which 67 are predicted to affect amino-acid sequence of one of the beta-defensin genes.

## Additional file

Additional file 1: Table S1. Frequency of rs200757797 and rs2740090 in the 1000 Genomes continental groups. (DOCX 13.4 kb)
