# Supplementary material for: Determining multiallelic complex copy number and sequence variation from high coverage exome sequencing data
Source: BMC Genomics. 2015 Nov 2;16:891. doi: 10.1186/s12864-015-2123-y (PMC4630827; doi:10.1186/s12864-015-2123-y)
Supplement: Additional file 1: Table S1. — Frequency of rs200757797 and rs2740090 in the 1000 Genomes continental groups. (DOCX 13.4 kb) [file 12864_2015_2123_MOESM1_ESM.docx]

**Table S1 Frequency of rs200757797 and rs2740090 in the 1000 Genomes continental groups**

| **Continental group** | **Frequency** | |
| --- | --- | --- |
|  | hg19:chr8:7679549  rs200757797  *DEFB105* C73Y | hg19:chr8:7754021  rs2740090  *DEFB4* P28P |
| **European** | 0.12 | 0.32 |
| **Sub-Saharan African** | 0.04 | 0.24 |
| **East Asian** | 0.19 | 0.42 |
| **South Asian** | 0.12 | 0.34 |
| **Admixed American** | 0.08 | 0.35 |
